# Unique Effects of Social Defeat Stress in Adolescent Male Mice on the Netrin-1/DCC Pathway, Prefrontal Cortex Dopamine and Cognition

**DOI:** 10.1523/ENEURO.0045-21.2021

**Published:** 2021-04-12

**Authors:** Philip Vassilev, Andrea Haree Pantoja-Urban, Michel Giroux, Dominique Nouel, Giovanni Hernandez, Taylor Orsini, Cecilia Flores

**Affiliations:** 1 Department of Psychiatry and Department of Neurology and Neurosurgery; 2 Integrated Program in Neuroscience, McGill University, Montréal, Quebec, H3A 2B4, Canada; 3 Douglas Mental Health University Institute, Montreal, Quebec, H4H 1R3, Canada

**Keywords:** adolescence, developmental biology, dopamine, guidance cues, prefrontal cortex, social defeat stress

## Abstract

For some individuals, social stress is a risk factor for psychiatric disorders characterized by adolescent onset, prefrontal cortex (PFC) dysfunction and cognitive impairments. Social stress may be particularly harmful during adolescence when dopamine (DA) axons are still growing to the PFC, rendering them sensitive to environmental influences. The guidance cue Netrin-1 and its receptor, DCC, coordinate to control mesocorticolimbic DA axon targeting and growth during this age. Here, we adapted the accelerated social defeat (AcSD) paradigm to expose male mice to social stress in either adolescence or adulthood and categorized them as “resilient” or “susceptible” based on social avoidance behavior. We examined whether stress would alter the expression of DCC and Netrin-1 in mesolimbic DA regions and would have enduring consequences on PFC DA connectivity and cognition. While in adolescence the majority of mice are resilient but exhibit risk-taking behavior, AcSD in adulthood leads to a majority of susceptible mice without altering anxiety-like traits. In adolescent, but not adult mice, AcSD dysregulates DCC and Netrin-1 expression in mesolimbic DA regions. These molecular changes in adolescent mice are accompanied by changes in PFC DA connectivity. Following AcSD in adulthood, cognitive function remains unaffected, but all mice exposed to AcSD in adolescence show deficits in inhibitory control when they reach adulthood. These findings indicate that exposure to AcSD in adolescence versus adulthood has substantially different effects on brain and behavior and that stress-induced social avoidance in adolescence does not predict vulnerability to deficits in cognitive performance.

## Significance Statement

During adolescence, dopamine (DA) circuitries undergo maturational changes which may render them particularly vulnerable to social stress. While social stress can be detrimental to adolescents and adults, it may engage different mechanisms and impact different domains, depending on age. The accelerated social defeat (AcSD) model implemented here allows exposing adolescent and adult male mice to comparable social stress levels. AcSD in adulthood leads to a majority of socially avoidant mice. However, the predominance of AcSD-exposed adolescent mice does *not* develop social avoidance, and these resilient mice show risk-taking behavior. Nonetheless, in adolescence only, AcSD dysregulates Netrin-1/DCC expression in mesolimbic DA regions, possibly disrupting mesocortical DA and cognition. The unique adolescent responsiveness to stress may explain increased psychopathology risk at this age.

## Introduction

Experiencing social stress in adolescence may be particularly harmful, since the adolescent period is characterized by ongoing maturational changes in the brain which can be disrupted by adverse experiences. A key adolescent maturational process is the gradual unfolding of the dopamine (DA) innervation to the prefrontal cortex (PFC; Kalsbeek et al., 1988; Voorn et al., 1988; Rosenberg and Lewis, 1995; Benes et al., 2000; Lambe et al., 2000; Manitt et al., 2011, 2013; Naneix et al., 2012; Willing et al., 2017; Hoops et al., 2018). Mesocorticolimbic DA axons reach the nucleus accumbens (NAcc) by early adolescence, and while some remain there (mesolimbic system), others (mesocortical system) continue growing to the PFC until early adulthood (Reynolds et al., 2018). The proper segregation of mesolimbic and mesocortical DA axons is regulated by the coordinated action of the guidance cue receptor DCC on DA axons and its ligand, Netrin-1, in the NAcc (Hoops and Flores, 2017; Hoops et al., 2018; Reynolds et al., 2018; Cuesta et al., 2020a).

Manipulations during adolescence that reduce DCC receptor expression in DA neurons or Netrin-1 in the NAcc cause mesolimbic DA axons to mistarget the NAcc and to instead grow ectopically to the PFC. This leads to changes in local synaptic organization within the medial PFC (mPFC) and to altered performance on cognitive tasks later in adulthood (Manitt et al., 2013; Reynolds et al., 2015, 2019). In humans, mounting evidence from postmortem (Manitt et al., 2013; Torres-Berrío et al., 2017) and genetic studies (Grant et al., 2012; Vosberg et al., 2018; Cross-Disorder Group of the Psychiatric Genomics Consortium, 2019; Strawbridge et al., 2019) shows that altered expression and polymorphisms in the *DCC* and *Netrin-1* genes associate with psychiatric disorders (for review, see Torres-Berrío et al., 2020; Vosberg et al., 2020). These disorders are linked to social stress and are characterized by PFC dysfunction, cognitive impairments and adolescent onset (McTeague et al., 2016). An important question is whether social stress in adolescence interferes with the Netrin-1/DCC pathway, disrupting the proper development of mPFC DA connectivity and ultimately causing cognitive impairments shared across multiple psychiatric disorders.

Rodent models of social stress represent a useful tool for addressing this question. A widely used paradigm is chronic social defeat stress (CSDS), where animals are exposed to repeated physical attacks and submission by an aggressive conspecific over 10 d (Krishnan et al., 2007; Miczek et al., 2008; Golden et al., 2011). As a result, some animals develop social avoidance (“susceptible”), while others do not (“resilient”). CSDS in adulthood leads to increased DA release in the PFC (Holly et al., 2015) and to changes in PFC DA receptor function that vary according to resilient and susceptible phenotype (Huang et al., 2016; Shinohara et al., 2018). Furthermore, optogenetic manipulation of mesolimbic and mesocortical DA projections alter adult social defeat stress susceptibility, but with opposite effects, suggesting fundamentally different roles of these two systems in social stress processing (Chaudhury et al., 2013). The mesocorticolimbic DA system is clearly involved in adult responses to stress, but much less is known about the impact of stress on this system during adolescence, when mesocortical innervation is still in progress. Social stress in adolescence may dysregulate the Netrin-1/DCC pathway and have unique consequences to mesocortical DA and cognitive development.

Here, we used accelerated social defeat (AcSD), a 4-d variation of the 10-d CSDS (Vialou et al., 2014; Sun et al., 2015; Heller et al., 2016), to expose adolescent and adult mice to comparable levels of social stress. The shorter duration of the AcSD paradigm allowed us to target stress exposure specifically to early adolescence when altering DCC levels in mesocorticolimbic DA neurons leads to profound neuroanatomical and behavioral changes in male mice (Yetnikoff et al., 2011, 2014; Manitt et al., 2013). We determine whether the molecular and behavioral consequences of social stress differ between early adolescence and adulthood.

## Materials and Methods

### Animals

Experimental procedures were performed in accordance with the guidelines of the Canadian Council of Animal Care and approved by the McGill University and Douglas Hospital Animal Care Committee. All mice were housed in a temperature-controlled and humidity-controlled (21–22°C; 60%) colony room of the Neurophenotyping center of the Douglas Mental Health University Institute, on a 12/12 h light/dark cycle (light on at 8 A.M.). The mice had *ad libitum* access to food and water throughout the experiments (except during food restriction for Go/No-Go experiments). Mice were assigned randomly to each experimental condition.

We tested adolescent [*n *=* *159, postnatal day (PND)25 at start of experiments] and adult (*n *=* *111, PND65 at start of experiments) male C57BL/6J wild-type mice (The Jackson Laboratory). These mice were housed in groups of four animals per cage before exposure to AcSD and single-housed after AcSD. Male CD-1 retired breeder mice (more than three months of age) obtained from Charles-River Canada were used as aggressors in the AcSD paradigm. CD-1 mice were used for a maximum of three consecutive experiments and for no longer than three months. These mice were single housed throughout the study.

### AcSD stress paradigm

#### Operational definition of “attack” in AcSD

In order to standardize procedures between experiments and experimenters, we used an operational definition of attack during AcSD sessions, defined as follows:

1. One attack is defined as “when the CD1 mouse bites the C57BL/6J mouse, and the C57BL/6J mouse moves away in response to the bite.”
2. A bite is defined as “when the CD1 mouse places its teeth on any part of the C57BL/6J mouse’s body.”
3. Moving away is defined as “when the C57BL/6J mouse moves both of its hind paws from the position they were in before the CD1 engaged it.”
4. There must be ∼2 s between separate attacks. If more than one bite occurs <2 s apart, count as one attack.
5. If the CD1 mouse bites more than once in succession without a break (<∼2 s apart), gently separate animals.
6. If the CD1 mouse bites and does not let go, gently separate animals.
7. If the C57BL/6J mouse becomes trapped in a corner or is pinned down by the CD1 mouse and cannot move away in response to a bite, gently separate animals with a ruler.
8. In cases 5–7, count as one attack (up to separation).

#### Adolescent AcSD, screening and priming for aggression

Before AcSD sessions, CD-1 mice were primed for aggressiveness in two phases. In the first phase, an adult (PND65) male C57BL/6 mouse was introduced to a CD-1 mouse’s home cage for 3 min or until it was attacked 10 times, whichever came first, on three consecutive days. On the fourth day, the second phase started, where priming was done twice a day (9:00 A.M. and 2:00 P.M.). In this phase, an adult C57BL/6 mouse was introduced to the home cage of the CD-1 mouse only for 30 s and then replaced by an adolescent (PND24–PND25) C57BL/6J mouse for 5 min. Phase 2 lasted 3–4 d until a subset of CD-1 mice became consistently aggressive toward the adolescent C57BL/6J mice. We recorded the latency and the number of attacks made by CD-1 mice toward adolescents in phase 2. Only CD-1 mice that attacked the C57BL/6J at least 10 times on at least two consecutive days were selected as aggressors for the AcSD procedure. The C57BL/6 adult and adolescent mice used for priming were not used for later experiments.

#### Adolescent AcSD, social defeat sessions

**Figure 1.**
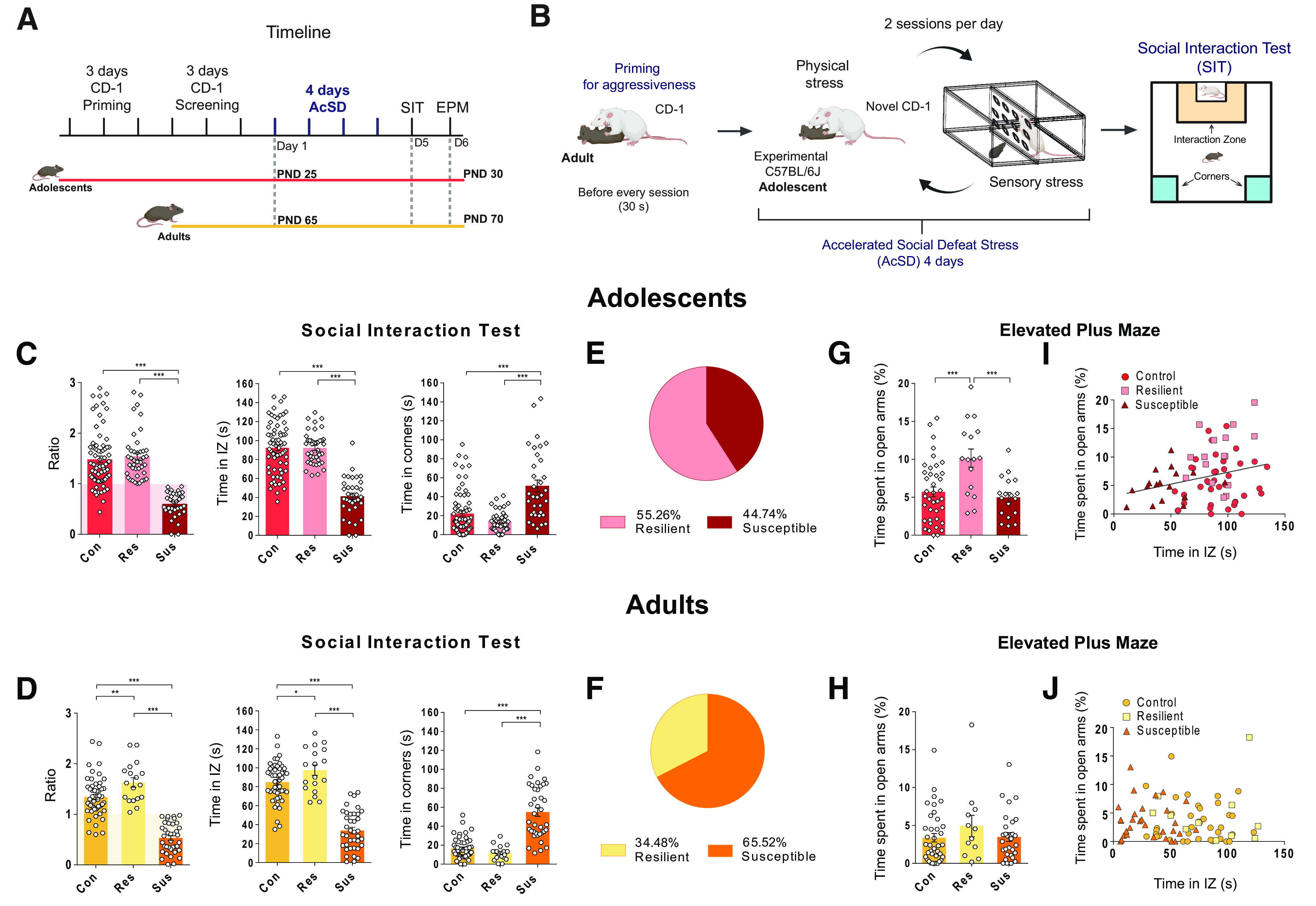
Differences in the resilience to AcSD stress in adolescence versus adulthood. The SIT data were pooled together from all experiments. ***A***, Experimental timeline. For AcSD in adolescence, CD-1 mice underwent two phases of priming and screening before the actual AcSD procedure. ***B***, Diagram representing the AcSD procedure. Only for AcSD in adolescence, CD-1 mice were primed with an adult C57BL/6 mouse for aggressiveness before each defeat session. ***C***, Behavior during the SIT after AcSD in adolescence. IZ, interaction zone; *** significantly different, *p *< 0.001. ***D***, SIT behavior following AcSD in adulthood; *** significantly different, *p *< .001; ** significantly different, *p *< 0.01; * significantly different, *p *< 0.05. ***E***, The majority of mice exposed to AcSD in adolescence are resilient, but (***F***) the majority of mice exposed to AcSD in adulthood are susceptible. ***G***, Following AcSD in adolescence, resilient mice spent more time in the open arms of the EPM, *** significantly different, *p* < 0.001. (***H***), AcSD in adulthood does not lead to changes in the EPM test. ***I***, the time spent in the open arms of the EPM and the time spent in the IZ during the SIT correlate significantly after AcSD in adolescence. ***J***, there is no correlation between the time spent in the open arms of the EPM and the time spent in the IZ after AcSD in adulthood. All data are shown as mean ± SEM.

The AcSD apparatus consisted of a transparent rat cage with two mouse housing compartments separated by a transparent and perforated central divider that allowed sensory but not physical contact between mice. Each selected CD-1 mouse previously screened for aggressive behavior was housed in one of the compartments for at least 48 h before the beginning of AcSD, to enhance the CD-1 mice’s territorial behavior. The other compartment remained empty. One day before the experiment, we repeated the procedure of priming phase 2 to ensure CD-1 mice were still aggressive toward adolescent C57BL/6J. The AcSD protocol consisted of two sessions/day (9:00 A.M. and 2:00 P.M.) for a total of 4 d (Covington et al., 2011; Vialou et al., 2014; Sun et al., 2015; Heller et al., 2016). Every session, an adult C57BL/6 mouse was introduced into the CD-1 compartment for a period of 30 s (to prime the CD-1 for aggressive behavior). Then, the adult C57BL/6 mouse was removed and an adolescent (PND25) C57BL/6J mouse was introduced as an intruder and left with the CD-1 until 10 min had passed or 10 attacks had occurred, whichever came first. At the completion of the session, the C57BL/6J adolescent mouse was housed overnight on the empty compartment to provide psychological stress (Fig. 1*B*). C57BL/6J adolescent mice were exposed to a new aggressor every day. Control C57BL/6J adolescent mice were housed in similar two-compartment rat cages with a conspecific every day. After the final AcSD session on day 4, all mice were re-housed individually for the remaining duration of the experiments.

##### Adult AcSD

The AcSD procedure for adult mice was nearly identical to the one described above for adolescent mice. The only difference was that there was no priming of CD-1 mice for aggression by introducing an additional C57BL/6 mouse in the beginning of screening and defeat sessions. There were only 3 d of screening for aggression where aggressive CD-1 mice were selected based on the same criterion as above: only CD-1 mice that attacked at least 10 times in 5 min on at least two consecutive days were selected as aggressors.

### Social interaction test (SIT)

The SIT was identical for both adolescent and adult mice. Twenty-four hours after the last session of AcSD, C57BL/6J adolescent or adult mice were assessed in the SIT to measure their approach and/or avoidance behavior toward a social target (Krishnan et al., 2007; Golden et al., 2011). Briefly, the SIT consisted of two sessions in which defeated and control mice were allowed to explore a squared-arena (42 × 42 cm) for a period of 2.5 min each session. In the first session, an empty wire mesh enclosure was located against one of the walls of the arena to determine baseline exploration. In the second session, an unfamiliar CD-1 aggressor was placed inside the wire mesh enclosure. The area that surrounded the enclosure was designated as the social interaction zone (14 × 9 cm), and the corners of the wall opposite to the enclosure were designated as corners (9 × 9 cm) and represented the farthest point from the social interaction zone. The time spent (in seconds) in the interaction zone and the corners was estimated during both sessions of the test. Animals that did not explore the arena during the first session (i.e., spent 0 s in any of the designated areas) were excluded from all analyses (2/159 adolescent mice). The social interaction ratio was calculated as the time spent in the interaction zone with the CD-1 aggressor present divided by the time spent in the interaction zone with the CD-1 aggressor absent. Defeated mice with a ratio <1.00 were classified as susceptible and a ratio ≥1.00 were classified as resilient. To ensure that high social interaction ratios reflected actual interest in the social target, resilient mice were also required to spend at least 60 s inside the interaction zone or were excluded from all analyses (5/159 adolescent and 2/111 adult mice). Mice with outlier interaction ratio scores were also excluded (4/159 adolescent and 2/111 adults). All SIT was performed under red light conditions between 11 A.M. and 4 P.M. and mice were tested in a counterbalanced order. Animal behavior was recorded with an overhead video camera for offline analysis using the software TopScanTM 2.0 (Clever Systems Inc.).

### Elevated plus maze (EPM)

On the day after the SIT, anxiety-like behavior was assessed in a plus-maze elevated 50 cm from the floor (Manitt et al., 2013; Torres-Berrío et al., 2017). The maze consisted of two facing open arms and two facing enclosed arms that extend from a central platform. C57BL/6J mice were placed in the center platform of the maze facing one of the open arms and left to explore the arms for 5 min. The time spent in the open versus closed arms of the maze were recorded with an overhead video camera for offline analysis using the software TopScanTM 2.0 (Clever Systems Inc.). The procedure was identical for adolescent (*n *=* *73) and adult (*n *=* *79) mice.

### Go/No-Go

To test cognitive function, we used the Go/No-Go task as previously described (Reynolds et al., 2018, 2019). Both adults (*n *=* *46) and adolescents (*n *=* *25) first went through AcSD and SIT; ∼40 d later mice were food restricted and started training for the behavioral task. Chocolate-flavored dustless precision pellets (BioServ) were used as a reinforcer. The task required the mice to nose poke in response to an illuminated “Go” cue within a limited amount of time or inhibit their response to this cue when presented together with an auditory “No-Go” cue. Nose poke responses to the Go cue (“hits”) resulted in the delivery of a food pellet, while the same response to the No-Go cue was considered a “commission error” and resulted in reward omission. High levels of commission errors were used as a measure of inhibitory control impairment. We also calculated a “correct response rate” which quantified the proportion of total available rewards that were obtained across both Go and No-Go trials (Cuesta et al., 2020b).

### Tissue dissection for Western blotting and quantitative PCR (qPCR)

Mice from the control, susceptible and resilient groups were euthanized by decapitation 7 d after the SIT. Brains were removed and flash frozen with 2-methylbutane chilled in dry ice. Bilateral punches of the pregenual mPFC [including prelimbic (PrL) and infralimbic (IL) subregions], the NAcc, and the ventral tegmental area (VTA) were taken from 1 mm-thick coronal sections, starting from plate 14 of the mouse brain atlas (Paxinos and Franklin, 2019) for mPFC and NAcc, and plate 55 for VTA.

### RNA extraction and qPCR

Total RNA fractions were isolated from the mouse frozen tissue (*n *=* *30 adolescents and *n *=* *26 adults) with the miRNeasy Micro kit protocol (QIAGEN). All RNA samples were determined to have 260/280 and 260/230 values ≥1.8, using the Nanodrop 1000 system (Thermo Scientific). Reverse transcription for *Dcc* mRNA was performed using High-Capacity cDNA Reverse Transcription kit (Applied Biosystems). qPCR (Reynolds et al., 2015; Torres-Berrío et al., 2017) using TaqMan assay (Applied Biosystems) was conducted with an Applied Biosystems QuantStudio 6 Flex Real-Time PCR system. Data for *Dcc* expression were analyzed by using the relative quantification method and *Gapdh* was used as reference gene. Real-time PCR was run in technical triplicates.

### Western blotting

As previously (Torres-Berrío et al., 2017; Cuesta et al., 2020a), protein samples (20 μg, *n *=* *25 adolescents and *n *=* *24 adults) were separated on a 10% SDS-PAGE and transferred to a PVDF membrane which was incubated overnight at 4°C with antibodies against Netrin-1 (Abcam, catalog #126729) and α-tubulin (Cell Signaling, catalog #2144S) for loading control. Four biological samples were included per experimental condition. Since Netrin-1 is a secreted protein which can be produced remotely (Brignani et al., 2020), we routinely quantify protein rather than mRNA levels.

### Neuroanatomical experiments

Mice (*n *=* *29 adolescents) were anesthetized with an overdose of a combination of ketamine 50 mg/kg, xylazine 5 mg/kg, acepromazine 1 mg/kg injected intraperitoneally, and perfused transcardially with 0.9% saline, followed by 4% PFA in PBS (pH 7.4). Coronal sections of the pregenual mPFC were obtained at 35 μm using a vibratome. Immunostaining was visualized with Alexa Fluor 594-conjugated secondary antibody raised in rabbit (Alexa Jackson).

### Stereology

As described previously (Manitt et al., 2013; Reynolds et al., 2018), the density of TH-positive (TH+) fibers and varicosities in the inner layers of the PrL and IL regions of the pregenual mPFC was evaluated using a stereological fractionator sampling design (West et al., 1991), with the optical fractionator probe of the Stereoinvestigator software (MicroBrightField). In the PFC, the TH antibody labels predominantly DA axons and rarely labels norepinephrine axons (Manitt et al., 2011, 2013). Regions of interest were delineated according to the mouse brain atlas (Paxinos and Franklin, 2019), and contours of the TH+ projection within these regions were traced at 5× magnification with a Leica DM4000B microscope. Stereoinvestigator calculates, for each brain region, a volume (in cubic micrometers) measurement from the contour area, section thickness, and section periodicity (MicroBrightField). Sections spanning plates 14–18 of the mouse brain atlas were studied. Stereoinvestigator calculates the total number of TH+ varicosities based on the experimenter’s random sampling of a known fraction of the region. Counting frame and grid size were chosen to consistently sample 33 sites per region.

### Experimental design and statistical analyses

The data for the molecular (qPCR and Western blotting), stereological and Go/No-Go experiments were gathered from separate cohorts of mice. All mice underwent the SIT, and the data from this test were pooled together from all cohorts. Mean values are presented with ±SEM. A significance threshold of α = 0.05 was used for all analyses. Data that were normally distributed and with similar variance across groups were analyzed with one-way and two-way ANOVAs. Significant main effects and/or interaction effects were followed by Holm–Sidak *post hoc* tests. Data that were not normally distributed or with heterogeneous variances were analyzed with a Kruskal–Wallis non-parametric test or a Brown–Forsythe’s ANOVA (respectively) followed by Dunn’s or Dunnett’s T3 *post hoc* tests. Outliers were identified using the ROUT method with a Q = 1%. The correlations were calculated using the Spearman or Pearson’s correlation coefficients (depending on whether the data were normally distributed) with two-tailed analysis, and a binomial test was used to compare the proportions of resilient and susceptible mice in adult and adolescent samples (using the proportions in one sample as expected values). All statistical tests were conducted using GraphPad Prism (GraphPad Software). Finally, area under the curve (AUC) was calculated using a custom MATLAB (MathWorks) script, available on request.

## Results

### Increased resilience to social defeat stress in adolescence versus adulthood

Figure 1*A* shows the experimental timeline, and Figure 1*B* illustrates the AcSD paradigm modified for use with adolescent mice. Following exposure to AcSD in adolescence, the SIT revealed a group of susceptible mice who were socially avoidant (social interaction ratios <1), and a resilient group (interaction ratios ≥1) who approached an unfamiliar social target similarly to control mice. Susceptible mice spent less time in the interaction zone during the SIT and more time in corner zones, away from the social target (interaction ratio, Kruskal–Wallis test, *H*(2) = 72.35, *p *<* *0.0001; Dunn’s *post hoc* tests: control vs susceptible, *p *<* *0.0001, control vs resilient, *p *>* *0.9999, resilient vs susceptible, *p *<* *0.0001; time spent in the interaction zone, Brown–Forsythe ANOVA, *F*_(2,129.2)_ = 78.80, *p *<* *0.0001; Dunnett’s T3 *post hoc* tests: control vs susceptible, *p *<* *0.0001, control vs resilient, *p *>* *0.9999, resilient vs susceptible, *p *<* *0.0001; time spent in the corner zones, Kruskal–Wallis test, *H*(2) = 32.45, *p *<* *0.0001, Dunn’s *post hoc* tests: control vs susceptible, *p *<* *0.0001, control vs resilient, *p *>* *0.9999, resilient vs susceptible, *p *<* *0.0001; Fig. 1*C*).

Mice exposed to AcSD in adulthood also segregated into susceptible and resilient phenotypes based on their interaction ratio scores and time spent in corner zones (interaction ratio, one-way ANOVA *F*_(2,104)_ = 79.57, *p *<* *0.0001, Holm–Sidak *post hoc* tests: control vs susceptible, *p *<* *0.0001, control vs resilient, *p *=* *0.004, resilient vs susceptible, *p *<* *0.0001; time spent in interaction zone, one-way ANOVA *F*_(2,104)_ = 85.95, *p *<* *0.0001; Holm–Sidak *post hoc* tests: control vs susceptible, *p *<* *0.0001, control vs resilient, *p *=* *0.03, resilient vs susceptible, *p *<* *0.0001; time spent in corners, Kruskal–Wallis test *H*(2) = 55.91, *p *<* *0.0001; Dunn’s *post hoc* tests: control vs susceptible, *p *<* *0.0001, control vs resilient, *p *=* *0.56, resilient vs susceptible, *p *<* *0.0001; Fig. 1*D*). These SIT data reveal a clear segregation between resilient and susceptible phenotypes in both adult and adolescent mice exposed to social defeat, in line with previous reports on AcSD and social avoidance (Vialou et al., 2014; Sun et al., 2015; Heller et al., 2016).

We used a standardized operational definition of an attack when exposing both adult and adolescent mice to social defeat. This allowed quantification and control of the amount of physical aggression received by adult and adolescent mice, and direct comparisons of effects between the two ages. Notably, the proportion of resilient animals was significantly higher when mice were exposed to AcSD in adolescence, indicating that, at this age, mice were less socially avoidant following social stress (55.26% vs 34.48%, respectively, Binomial test *p *=* *0.0002; Fig. 1*E*,*F*).

Since, on some occasions, adolescent mice received slightly fewer attacks compared with adults (as defined by our operational definition of an attack), we addressed whether physical harm alone could account for the age differences in the proportion of resilient/susceptible phenotypes. We used our attack records from adolescent mice to calculate (1) the percentage of animals attacked <10 and 0 times on each defeat session; (2) the average of these measures across all sessions; and (3) the average number of attacks received by adolescent mice across all defeat sessions. Using these measures, we reproduced the “adolescent” attack pattern in a separate adult cohort (*n *=* *15). We also *increased* the number of attacks in a cohort of adolescent mice by allowing up to 15 instead of 10 attacks for some defeat sessions. Even after these manipulations, there were still more resilient mice following AcSD in adolescence versus adulthood (60% vs 33.3%, respectively, one-tailed Binomial test, *p *=* *0.03; Extended Data Fig. 1-1*B*). There were no significant correlations between the number of attacks received and the time spent with the social target in the SIT (adolescent, all cohorts, Spearman’s *r*(52) = −0.19, *p *=* *0.18; adolescent, increased attacks, Pearson’s *r*(15) = –0.06, *p *=* *0.02, *R*^2^ = 0.004; adult, reduced attacks, Pearson’s *r*(14) = 0.38, *p *=* *0.19, *R*^2^ = 0.14; Extended Data Fig. 1-1*C*).

10.1523/ENEURO.0045-21.2021.f1-1 Extended Data Figure 1-1 ***A***, Proportion of resilient and adolescent animals as a proportion of all adult and adolescent cohorts (as in Fig. 1). ***B***, The proportions of resilient and susceptible animals following AcSD in adulthood or adolescence did not change significantly after adjusting the number of attacks. The majority of mice exposed to AcSD in adolescence were resilient even after increasing the number of attacks received. The majority of mice exposed to AcSD in adulthood were susceptible even after reducing the number of attacks received. ***C***, There were no significant correlations between the average number of attacks received and the time spent in the interaction zone during the SIT. Download Figure 1-1, EPS file.

Another difference between AcSD in adolescence versus adulthood was mouse behavior in the EPM. Following AcSD in adolescence, resilient mice spent significantly more time in the open arms of the EPM relative to control and susceptible groups (one-way ANOVA *F*_(2,70)_ = 9.01, *p *<* *0.001, Holm–Sidak *post hoc* tests: resilient vs susceptible and control *p *<* *0.001; Fig. 1*G*). This suggests a higher propensity for risk-taking-like behaviors in adolescent resilient mice, given the natural propensity of rodents to avoid spaces which are exposed to attacks from predators. Indeed, the time spent inside the interaction zone during the SIT was positively correlated with the time spent in the EPM open arms (Pearson’s *r*(73) = 0.26, *p *= 0.02, *R*^2^ = 0.07; Fig. 1*I*). In adult AcSD-exposed mice, there was no relationship between stress phenotype and EPM performance (Kruskal–Wallis test, *H*(2) = 1.31, *p *= 0.52; Fig. 1*H*) and the time spent in the open arms and in the interaction zone did not correlate (Pearson’s *r*(73) = −0.01, *p *=* *0.92; Fig. 1*J*). The increased resilience of adolescent mice to AcSD may be explained by a general predisposition to risk-taking.

### Unique effect of social defeat stress in adolescence on the Netrin-1/DCC pathway of the mesolimbic DA system

**Figure 2.**
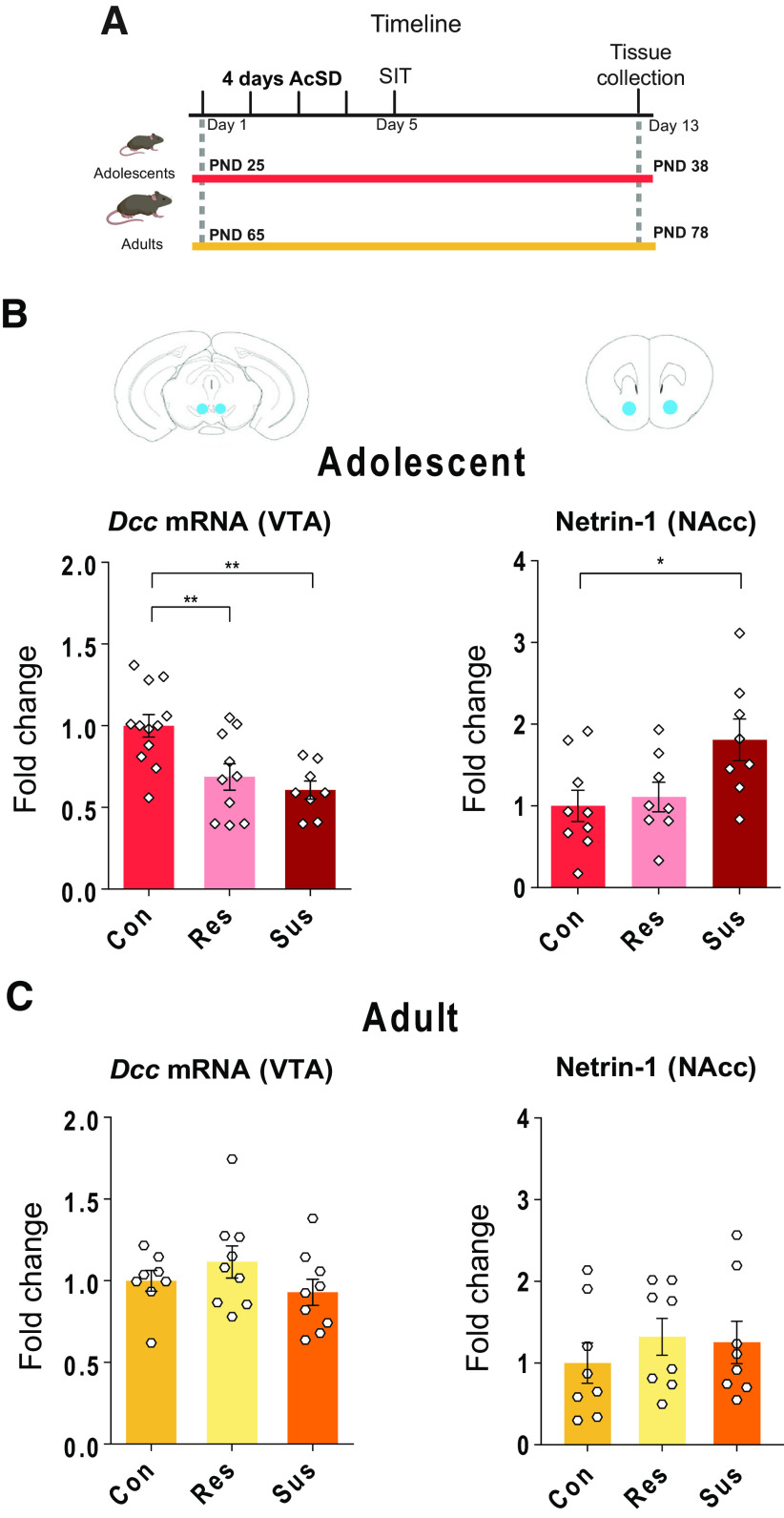
AcSD in adolescence, but not adulthood, dysregulates the Netrin-1/DCC pathway in the mesolimbic DA system. ***A***, Experimental timeline. ***B***, AcSD in adolescence leads to downregulation of *Dcc* mRNA expression in the VTA of both resilient and susceptible mice, while Netrin-1 protein levels in the NAcc are upregulated only in the susceptible group; ** significantly different, *p* < 0.01; * significantly different, *p* < 0.05. ***C***, AcSD in adulthood does not lead to changes in *Dcc* mRNA expression in the VTA or Netrin-1 protein levels in the NAcc. All data are shown as mean ± SEM.

To determine whether AcSD in adolescence dysregulates the Netrin-1/DCC pathway, we measured *Dcc* mRNA expression in the VTA and Netrin-1 protein levels in the NAcc one week after AcSD (Fig. 2*A*). There was reduced expression of *Dcc* mRNA in the VTA of both resilient and susceptible mice, indicating that social defeat in adolescence alters VTA *Dcc* expression independently of social avoidance. However, in the NAcc, Netrin-1 protein levels were elevated in susceptible but not resilient mice (VTA *Dcc*, one-way ANOVA *F*_(2,27)_ = 8.76, *p *=* *0.001, Holm–Sidak *post hoc* tests: resilient vs control, *p *=* *0.007, susceptible vs control, *p *=* *0.002, resilient vs susceptible, *p *=* *0.46; NAcc Netrin-1, one-way ANOVA *F*_(2,22)_ = 4.310, *p *=* *0.03, Holm–Sidak *post hoc* tests: susceptible vs control, *p *=* *0.04, resilient vs control, *p *=* *0.71, resilient vs susceptible, *p *=* *0.06; Fig. 2*B*). Since the complementary actions of DCC receptors on axons of VTA DA neurons and Netrin-1 in the NAcc dictate the segregation of mesolimbic and mesocortical projections (Manitt et al., 2013; Reynolds et al., 2018; Cuesta et al., 2020a), the downregulation of VTA *Dcc* mRNA in resilient mice suggests targeting errors of mesolimbic DA axons and concomitant changes in adult PFC DA connectivity. The upregulation of NAcc Netrin-1, in susceptible mice, suggests a compensatory effect.

The levels of *Dcc* in the VTA did not differ between resilient, susceptible and control animals following AcSD in adulthood, nor did Netrin-1 levels in the NAcc (VTA *Dcc*, Kruskal–Wallis, *H*(2) = 2.38, *p *=* *0.3; NAcc Netrin-1, one-way ANOVA *F*_(2,21)_ = 0.4816, *p *=* *0.62; Fig. 2*C*). This is in line with our previous reports of no change in VTA *Dcc* expression after 10-d CSDS in adulthood (Torres-Berrío et al., 2017).

### Social defeat stress in adolescence disrupts the extent and organization of PFC DA connectivity in adulthood

**Figure 3.**
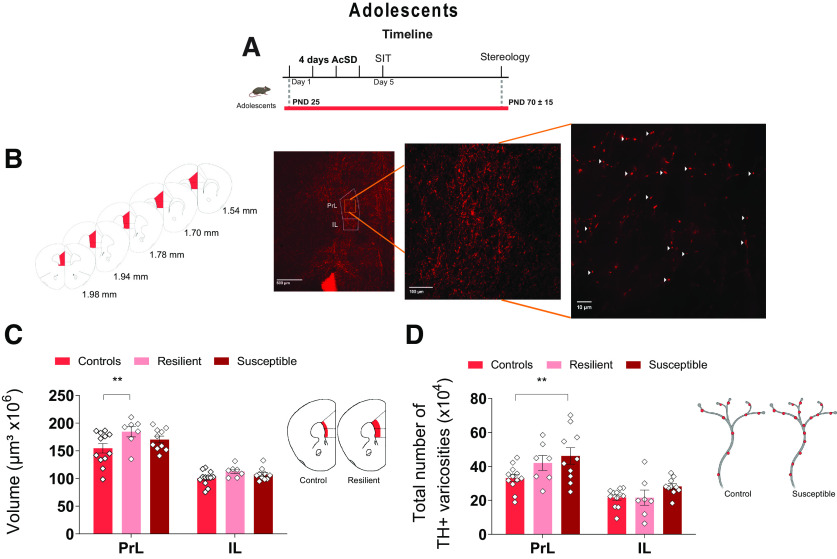
Exposure to AcSD in adolescence leads to altered mPFC DA connectivity in adulthood. ***A***, Experimental timeline. ***B***, Coronal sections containing the sampled PrL and IL mPFC subregions. The photomicrographs show TH-immunolabeled DA axons in deep PrL and IL layers (5×, 20×, and 100× magnifications). White arrows indicate examples of TH+ varicosities. ***C***, AcSD in adolescence leads to increased expanse of the DA innervation to the PrL cortex of resilient mice; ** significantly different, *p *< 0.01; and to (***D***) increased total number of DA varicosities in the PrL cortex of susceptible mice; ** significantly different, *p* < 0.01. All data are shown as mean ± SEM.

To assess whether the effects of adolescent AcSD on the Netrin-1/DCC pathway translate into changes in PFC DA connectivity, we quantified the extent of the DA innervation to the PrL and IL portions of the mPFC of adult mice that were exposed to AcSD in adolescence (Fig. 3*A*,*B*).

There was an overall increase in the volume that DA fibers occupy across the PrL and IL cortices of resilient, but not susceptible mice. This finding is consistent with the decrease in VTA *Dcc* expression in resilient mice, and the presumably compensatory increase in NAcc Netrin-1 in the susceptible group (two-way ANOVA, main effect of phenotype, *F*_(2,52)_ = 5.17, *p *=* *0.009, main effect of region, *F*_(1,52)_ = 139.3, *p *<* *0.0001, interaction, *F*_(2,52)_ = 0.90, *p *= 0.41; Holm–Sidak *post hoc* tests, PrL: resilient vs control, *p *=* *0.008, susceptible vs control, *p *=* *0.13, resilient vs susceptible, *p *=* *0.16; Fig. 3*C*).

Susceptible, but not resilient, mice showed a significant increase in the total number of mPFC DA varicosities relative to controls, suggesting stress-induced alterations in fine axonal architecture (two-way ANOVA, main effect of phenotype, *F*_(2,52)_ = 5.47, *p *=* *0.007, main effect of region, *F*_(1,52)_ = 40.05, *p *<* *0.0001, interaction, *F*_(2,52)_ = 1.109, *p *=* *0.34; Holm–Sidak *post hoc* tests, PrL: susceptible vs control, *p *=* *0.009, resilient vs control, *p *=* *0.12, resilient vs susceptible, *p *=* *0.39; Fig. 3*D*). Overall, social defat stress in adolescence alters adult mPFC DA connectivity, with the precise type of changes differing between resilient and susceptible animals.

### Social defeat stress in adolescence, but not adulthood, has enduring detrimental consequences for cognition

**Figure 4.**
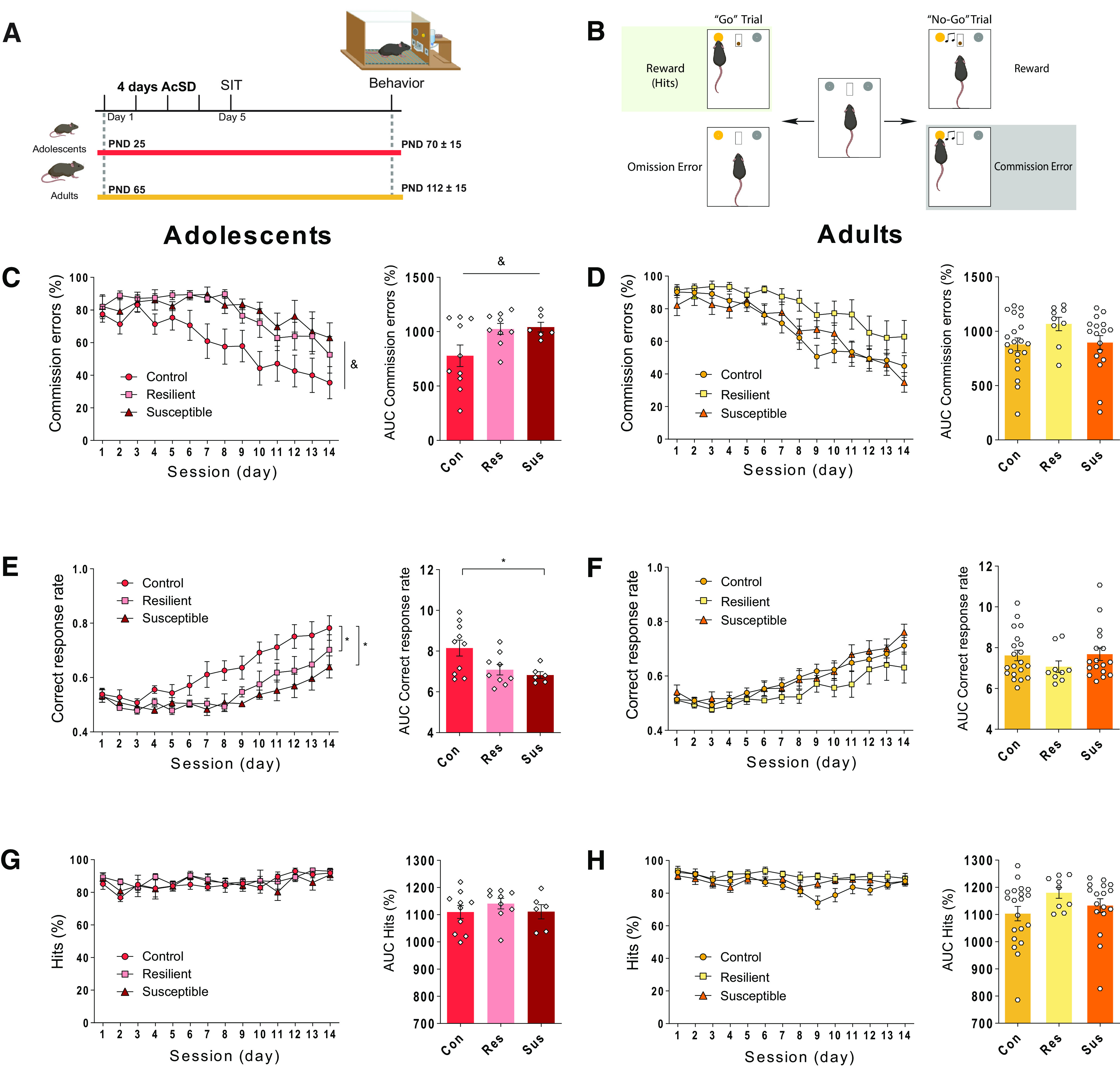
AcSD in adolescence, but not adulthood, leads to deficits in inhibitory control in adulthood. ***A***, Experimental timeline. ***B***, Go/No-Go task. ***C***, AcSD in adolescence leads to an increase in proportion of commission errors in defeated mice, regardless of behavioral phenotype shown in the SIT (Fig. 1); & significant main effect of phenotype, *p *< 0.05. ***D***, AcSD in adulthood does not alter inhibitory control six weeks later. ***E***, Correct response rate representing the proportion of total rewards acquired from both Go and No-Go trials. AcSD in adolescence leads to decreased correct response rate in resilient and susceptible mice relative to controls; * significantly different, *p *< 0.05. ***F***, AcSD in adulthood does not alter correct response rate. There are no significant differences in hits between groups following AcSD (***G***) in adolescence (***H***) or in adulthood.

To assess whether AcSD in adolescence alters cognitive processing in adulthood, we tested mice on the Go/No-Go task (Fig. 4*A*,*B*). Both resilient and susceptible mice were impaired on the task as evident by a significant main effect of phenotype and a higher proportion of commission errors on average (two-way ANOVA, main effect of phenotype, *F*_(2,22)_ = 3.66, *p *=* *0.04; Holm–Sidak *post hoc* tests: control vs resilient, *p *=* *0.06, control vs susceptible, *p *=* *0.06; main effect of day, *F*_(13,286)_ = 12.94, *p *< 0.0001, interaction effect, *F*_(26,286)_ = 1.27, *p *= 0.18; AUC, Brown-Forsythe ANOVA *F*_(2,15.97)_ = 4.68, *p* = 0.02; Dunnett’s T3 *post hoc* tests: control vs resilient, *p *=* *0.09, control vs susceptible, *p *=* *0.06; Fig. 4*C*). This impairment was also evident when considering the overall correct response rate which was lower for resilient and susceptible groups (two-way ANOVA, main effect of phenotype, *F*_(2,22)_ = 4.51, *p *=* *0.02; Holm–Sidak *post hoc* tests: control vs resilient, *p *=* *0.03, control vs susceptible *p *=* *0.03; main effect of day, *F*_(13,286)_ = 20.80, *p *<* *0.0001, interaction effect, *F*_(26,286)_ = 1.58, *p *=* *0.04; AUC, Brown–Forsythe ANOVA *F*_(2,17.6)_ = 5.89, *p *=* *0.01; Dunnett’s T3 *post hoc* tests: control vs resilient, *p *=* *0.07, control vs susceptible, *p *=* *0.02; Fig. 4*E*). Thus, although resilient mice show protection against the immediate effect of social defeat stress on social avoidance, they are vulnerable to the long-term negative consequences of AcSD on cognition. There were no significant group differences in the proportion of hits, suggesting that all mice were similarly engaged in the task, and that differences in correct response rate reflected mainly differences in commission errors (two-way ANOVA, main effect of phenotype, *F*_(2,22)_ = 0.59, *p *=* *0.56; main effect of day, *F*_(13,286)_ = 3.03, *p *=* *0.0003, interaction effect, *F*_(26,286)_ = 0.83, *p *=* *0.71; AUC, *F*_(2,22)_ = 0.5847, *p *=* *0.57; Fig. 4*G*).

Following exposure to AcSD in adulthood, there were no significant differences between control, resilient or susceptible mice in inhibitory control, i.e., there were no significant differences in the proportion of commission errors (two-way ANOVA, main effect of phenotype, *F*_(2,43)_ = 1.89, *p *=* *0.16; main effect of day, *F*_(13,559)_ = 35.23, *p *< 0.0001, interaction effect, *F*_(26,559)_ = 1.59, *p *=* *0.03; AUC, Kruskal–Wallis test, *H*(2) = 4.48, *p *=* *0.11; Fig. 4*D*) or in correct response rate (two-way ANOVA, main effect of phenotype, *F*_(2,43)_ = 1.00, *p *=* *0.38; main effect of day, *F*_(13,559)_ = 33.97, *p *<* *0.0001, interaction effect, *F*_(26,559)_ = 0.94, *p *=* *0.54; AUC, Kruskal–Wallis test, *H*(2) = 2.48, *p *=* *0.29; Fig. 4*F*). There were also no differences between groups in the average number of hits, suggesting similar task engagement (two-way ANOVA, main effect of phenotype, *F*_(2,43)_ = 1.54, *p *=* *0.23; main effect of day, *F*_(13,559)_ = 3.22, *p *=* *0.0001, interaction effect, *F*_(26,559)_ = 1.73, *p *= 0.01; AUC, Kruskal–Wallis test, *H*(2) = 3.67, *p *=* *0.16; Fig. 4*H*).

## Discussion

Exposure to AcSD in adolescence versus adulthood has different short and long-term effects on brain and behavior of male mice. Following AcSD in adolescence, the majority of mice do not develop social avoidance, and these resilient mice exhibit risk-taking behavior. Instead, AcSD in adulthood leads to a majority of socially avoidant (i.e., susceptible) mice and does not lead to anxiety-like traits. AcSD in adolescence, but not adulthood, dysregulates the Netrin-1/DCC pathway in the VTA and NAcc. The pattern of this dysregulation differs between susceptible and resilient mice and is associated with specific changes in DA connectivity in the PrL subregion of the mPFC. Mice exposed to AcSD in adolescence show a cognitive impairment in the Go/No-Go task when they have reached adulthood, while mice exposed to AcSD in adulthood remain unaffected.

### Social defeat stress in adolescence dysregulates the Netrin-1/DCC pathway and impacts PFC DA connectivity

The downregulation of *Dcc* mRNA in the VTA and the increase in the expanse of DA fibers within the PrL cortex of resilient mice are consistent with the ectopic growth of mesolimbic DA axons to the mPFC in mice with reduced *Dcc* expression in adolescence (Manitt et al., 2013; Reynolds et al., 2018). Social stress in adolescence may disrupt mesocorticolimbic DA system maturation by causing targeting errors in growing DA axons. In susceptible mice, although there is a reduction in *Dcc* expression in the VTA, DA fiber volume within the PrL cortex is not significantly different from control groups. This may be explained by the selective and likely compensatory increase of Netrin-1 expression in the NAcc, which may counteract DA axon mistargeting. We cannot conclude if the changes in the Netrin-1/DCC pathway account for the immediate response to stress (resilience vs susceptibility) or if they are causally linked to changes in PFC DA innervation and cognitive behavior long after adolescent stress exposure, in adulthood. These questions will be directly addressed by genetically manipulating *Dcc* expression in VTA DA neurons and Netrin-1 in the NAcc. However, our findings from adult male mice using the 10-d CSDS paradigm indicate that resilience and susceptibility depend on *Dcc* changes in pyramidal neurons of the PFC, and not *Dcc* in DA neurons of the VTA (Torres-Berrío et al., 2017). The increased DA varicosity number in susceptible mice may reflect other mechanisms of stress-induced anatomic changes in addition to or instead of those involving the Netrin-1/DCC pathway in the NAcc. One possibility is abnormal synapse formation or pruning since the vast majority of DA varicosities in the PFC make synaptic connections (Séguéla et al., 1988). It remains to be determined whether these anatomic alterations are specific to adolescents and whether distinct mechanisms (i.e., beyond changes in DCC/Netrin-1 signaling) underlie changes in susceptible versus resilient mice.

### Social stress in adolescence may disrupt inhibitory control in adulthood through changes in PFC DA signaling

DA signaling in the PFC plays an important role in cognitive function and PFC DA axons make highly specific connections within functionally distinct PFC subdivisions (for review, see Tritsch and Sabatini, 2012). Subtle changes in PFC DA connectivity following AcSD in adolescence may be linked to cognitive impairments observed in adulthood. For example, DA release in the mPFC is positively correlated with performance on a set-shifting task (Stefani and Moghaddam, 2006). However, excessive DA release during this task can lead to perseverance errors (Ellwood et al., 2017), suggesting that optimal performance requires highly-specific DA release dynamics rather than simply an overall increase in mPFC DA. PFC DA is also involved in assigning salience: pairing of DA release in the mPFC with conditioned stimuli improves their subsequent discrimination and biases responding toward them (Popescu et al., 2016). The Go/No-Go task which we have used to assess inhibitory control involves cue discrimination (e.g., Go vs No-Go signals) and quantifies perseverance-like errors (e.g., responding on No-Go trials). Cognitive impairment in adult mice exposed to social defeat in adolescence may result from changes in mPFC DA connectivity and concomitant alterations in extracellular DA (Reynolds et al., 2019). The simplest prediction based on the increase of DA fiber volume and varicosity number following AcSD in adolescence would be that adult extracellular DA levels will increase. We find apparently distinct anatomic alterations in resilient versus susceptible mice, which may occur through different mechanisms. If volumetric changes in resilient mice result from ectopic growth of mesolimbic DA axons to the PFC, then the net result on DA release dynamics may be more complex because of the electrophysiological and molecular differences between mesolimbic and mesocortical DA projections (Lammel et al., 2008). Indeed, others have shown that adult rats exposed to social stress in adolescence have reduced extracellular DA in the IL cortex because of increased DA transporter function and DA D2 autoreceptor activity (Novick et al., 2015; Weber et al., 2018). We are investigating whether ectopic growth of mesolimbic DA axons to the PFC changes its temporal dynamics of DA release.

### Increased resilience to stress-induced social avoidance in adolescence does not protect against cognitive impairments in adulthood

We found a higher proportion of resilient mice following AcSD in adolescence versus adulthood, even after controlling for the number of received attacks. This difference may be explained by several factors including a higher propensity to explore a social target (Panksepp et al., 2007; Fairless et al., 2012, 2013) and a lower corticosterone response to stress in adolescent mice (Adriani and Laviola, 2000; Montagud-Romero et al., 2015, 2017). The more risk-taking phenotype in the EPM in adolescent resilient mice may explain their response to the potential threat of the social target in the SIT. However, resilience to social avoidance following AcSD in adolescence did not confer resilience to adult cognitive deficits: both resilient and susceptible mice were impaired on the Go/No-Go task. This is very intriguing considering that many of the negative metabolic, neurobiological and behavioral consequences of social defeat stress in adulthood are specific to socially avoidant (susceptible) mice (Krishnan et al., 2007), including changes in mesocorticolimbic DA signaling (Chaudhury et al., 2013; Bagot et al., 2015; Shinohara et al., 2018). Here, we show that AcSD, specifically in adolescence, produces inhibitory control impairment independently from social avoidance. Both impaired inhibitory control (impulsivity) and high risk-taking are traits associated with addiction vulnerability (Egervari et al., 2018), and we find increased risk-taking behavior in adolescent resilient mice. One possibility is that AcSD in adolescence leads to an addiction-prone phenotype in some mice which manifests as resilient during the SIT but shows impairment in the Go/No-Go task.

Our findings on the effect of AcSD on EPM behavior appear to be specific to the social defeat procedure we have employed. Previous studies have reported that exposure to social defeat leads to anxiety-like behavior in the EPM in adult animals (Krishnan et al., 2007; Duque et al., 2017; Torres-Berrío et al., 2017; Blanco-Gandia et al., 2019) as well as adolescents (Iñiguez et al., 2014). However, all these studies have used longer protocols with 10–20 sessions of social defeat or have exposed mice at later adolescent ages (e.g., PND30+). Both the duration of the stress exposure (Musazzi et al., 2017) and the age of the subjects (Cotella et al., 2019) may explain the differences between EPM behavior in this study and others.

### The effect of social defeat stress on the Netrin-1/DCC pathway is age and region specific

AcSD in adulthood does not affect expression of *Dcc* mRNA in the VTA or of Netrin-1 in the NAcc, consistent with our previous report showing no changes in *Dcc* expression in the VTA following adult exposure to CSDS (Torres-Berrío et al., 2017). Notably, 10 d of CSDS in adult mice leads to upregulation of *Dcc* mRNA in the PFC, and this effect is necessary for developing susceptibility (Torres-Berrío et al., 2017). Thus, social stress regulates the Netrin-1/DCC pathway with age and brain region specificity, consistent with the distinct spatiotemporal role of this pathway in the organization of developing and matured neuronal networks (Morgunova et al., 2020; Torres-Berrío et al., 2020). We have observed similar spatiotemporal specificity following exposure to stimulant drugs of abuse: while amphetamine (at doses corresponding to recreational use) downregulates *Dcc* mRNA expression in the VTA in early adolescence, the same treatment does not alter VTA transcript levels in mid-adolescence and upregulates them on adult exposure (Yetnikoff et al., 2010, 2011, 2014). Recreational-like doses of amphetamine also disrupt adult PFC DA connectivity and inhibitory control in mice exposed during early, but not mid-adolescence or adulthood (Reynolds et al., 2019). Since both social stress and drugs of abuse affect the Netrin-1/DCC pathway in an age-specific and region-specific manner, it is likely that this specificity extends to other environmental factors. An important question is whether the sensitivity of the Netrin-1/DCC pathway is also sex-specific, considering sex-differences in adolescent brain development (Lenroot and Giedd, 2010), prevalence of mood disorders (Seney and Sibille, 2014) and impulsivity (Weafer and de Wit, 2014). We now have preliminary evidence that our AcSD procedure is applicable to female adolescent mice as well, and we are currently working on addressing the question of sex differences in the effect of AcSD on the Netrin-1/DCC pathway, PFC DA development and inhibitory control.

### A distinct role for DCC in adolescence and adulthood

We have previously reported that upregulation of the DCC receptor in pyramidal neurons of the PFC in adult male mice confers susceptibility to stress in the 10-d CSDS paradigm (Torres-Berrío et al., 2017). In the present study we developed a novel procedure to expose male mice to social stress in early adolescence and study *Dcc* expression in DA neurons of the VTA. Our aim was to assess the effects of stress on the Netrin-1/DCC pathway within the mesocorticolimbic system in light of our previous reports on early adolescence as a critical developmental period for DA innervation to the PFC. An important distinction to make between our adult and adolescent studies is that the role of *Dcc* changes as a function of age. In adolescence, changes in *Dcc* can result in large scale changes in neuronal connectivity since DA axons are still traveling long distances to find their targets. Our amphetamine studies show that this results in abnormal DA innervation to the PFC and impaired cognition later in adulthood and here we show that these effects may apply to the case of social stress as well. In all our adult experiments we focus on the role of *Dcc* in the matured PFC and in the possible role of its protein in reorganization of local connectivity.

In conclusion, social defeat stress in adolescence leads to a constellation of molecular, anatomic and behavioral changes which suggest abnormal mesocorticolimbic DA development. The effects of social defeat on Netrin-1/DCC expression indicate that this guidance cue system serves as a common substrate for PFC dysfunction caused by adverse environmental factors in adolescence. Since impairments in PFC function are characteristic of multiple psychiatric disorders, the Netrin-1/DCC pathway may represent a critical target for early intervention and treatment strategies. The distinct effects of social stress on the Netrin-1/DCC pathway, social behavior, and cognition between adolescence and adulthood further highlight that adolescence is a unique period for vulnerability and resilience to mental illness and indicate that environmental factors change the developing and the matured brain through fundamentally different mechanisms.

## Synthesis

Reviewing Editor: Carmen Sandi, Swiss Federal Institute of Technology Decisions are customarily a result of the Reviewing Editor and the peer reviewers coming together and discussing their recommendations until a consensus is reached. When revisions are invited, a fact-based synthesis statement explaining their decision and outlining what is needed to prepare a revision will be listed below. Note: If this manuscript was transferred from JNeurosci and a decision was made to accept the manuscript without peer review, a brief statement to this effect will instead be what is listed below.

This revised manuscript version has satisfactorily addressed the issues previously raised by two reviewers. The study represents an important addition to the field of stress and neurodevelopmental changes by illustrating how a critical window during adolescence is susceptible to stress (i.e., an accelerated social defeat procedure) affects behavioral measures, gene expression, and dopaminergic innervation of limbic brain regions. The study presents compelling data in support of the hypothesis that adolescent social stress exerts long-lasting effects through disruption of dopaminergic axon growth. The study builds on the lab’s extensive previous work showing that dopamine neurons need DCC-Netrin1 signaling, specifically during adolescence, to properly target NAcc, and show here that stress during adolescence can disrupt these processes. Specifically, this study shows that acute social stress alters expression of DCC (in VTA) and Netrin-1 (in NAcc, only in behaviorally susceptible mice). The study also shows that social stress, specifically in adolescence, alters dopaminergic innervation of PrL. Furthermore, adolescent social stress, but not adult social stress, was shown to alter cognitive behaviors 6-7 weeks after stress. Overall, this study advances our understanding of the neurobiological mechanisms whereby stress experienced during adolescence affect specific aspects of the dopaminergic system.
